# Increased susceptibility of airway epithelial cells from ataxia-telangiectasia to *S. pneumoniae* infection due to oxidative damage and impaired innate immunity

**DOI:** 10.1038/s41598-019-38901-3

**Published:** 2019-02-22

**Authors:** Abrey J. Yeo, Anna Henningham, Emmanuelle Fantino, Sally Galbraith, Lutz Krause, Claire E. Wainwright, Peter D. Sly, Martin F. Lavin

**Affiliations:** 1 Neuroscience & Infectious Disease Group, The University of Queensland Centre for Clinical Research, Herston, Queensland Australia; 2 Children’s Lung, Environment and Asthma Research (CLEAR) Group, Child Health Research Centre, The University of Queensland, South Brisbane, Queensland Australia; 3 The University of Queensland Diamantina Institute, Translational Research Institute, Woolloongabba, Queensland Australia; 4 Lady Cilento Children’s Hospital, South Brisbane, Queensland Australia

## Abstract

Respiratory disease is a major cause of morbidity and mortality in patients with ataxia-telangiectasia (A-T) who are prone to recurrent sinopulmonary infections, bronchiectasis, pulmonary fibrosis, and pulmonary failure. Upper airway infections are common in patients and *S. pneumoniae* is associated with these infections. We demonstrate here that the upper airway microbiome in patients with A-T is different from that to healthy controls, with *S. pneumoniae* detected largely in patients only. Patient-specific airway epithelial cells and differentiated air-liquid interface cultures derived from these were hypersensitive to infection which was at least in part due to oxidative damage since it was partially reversed by catalase. We also observed increased levels of the pro-inflammatory cytokines IL-8 and TNF-α (inflammasome-independent) and a decreased level of the inflammasome-dependent cytokine IL-β in patient cells. Further investigation revealed that the ASC-Caspase 1 signalling pathway was defective in A-T airway epithelial cells. These data suggest that the heightened susceptibility of these cells to *S. pneumoniae* infection is due to both increased oxidative damage and a defect in inflammasome activation, and has implications for lung disease in these patients.

## Introduction

Ataxia-telangiectasia (A-T) is an autosomal recessive disorder with an estimated incidence of 1 in 100,000^1,2^. It is a multisystem disease characterized by neurodegeneration, recurrent sinopulmonary infection, immunodeficiency, lung disease, radiosensitivity, chromosomal instability, sterility, and cancer susceptibility^3,4^. Lung disease associated with chronic sinopulmonary infection, bronchiectasis, and interstitial lung changes is common in patients with A-T and is responsible for significant morbidity and mortality^5–7^. Pulmonary infections in A-T are usually caused by common bacterial pathogens such as *Haemophilus influenzae*, *Streptococcus pneumoniae*, *Pseudomonas aeruginosa* and *Staphylococcus aureus*^5,6,8,9^. Hence it is crucial for patients to receive appropriate respiratory management after they have been diagnosed with this disorder. It has been established that a number of factors contribute to the onset of pulmonary abnormalities including susceptibility to infection, abnormal immune responses, recurrent aspiration and impaired clearance of respiratory tract secretions^8,9^. However, the exact basis of the susceptibility to lung disease in patients with A-T remains unknown. Current treatment for respiratory symptoms is largely based on extrapolation from that employed with other more common disorders such as cystic fibrosis and primary immunodeficiencies^9,10^.

A-T is caused by mutations in the ataxia-telangiectasia mutated (ATM) gene. The ATM protein plays a central role in DNA double strand break repair and signalling, oxidative stress response and other cellular processes^4,11^.We and others have previously provided evidence that oxidative stress contributes to the A-T phenotype in cells in culture^12,13^, in animal models^14–16^ and in patient-derived stem cells^17,18^. Previous reports have shown that mammalian cells lacking ATM display high concentrations of reactive oxygen species (ROS) and hypersensitivity to agents that induce oxidative stress^12^. It has also been shown that the increased incidence of lymphoma and the loss of hematopoietic stem cells that occur in Atm-deficient mice can be suppressed by antioxidants^19–21^. These data thus point to an important role for ATM in regulating cellular defences against oxidative stress. We now propose that oxidative stress is likely to be a contributing factor in initiation and progression of lung disease. Many of the bacterial species that infect the respiratory tract and that are associated with progressive lung disease, including *Streptococcus pneumoniae*^9^, produce hydrogen peroxide (H_2_O_2_), a strong oxidizing agent^22^. We have previously demonstrated that the ATM kinase is activated by H_2_O_2,_ that generates ROS, by a mechanism that is distinct from that caused by DNA double strand breaks (DSB), providing evidence that it is central to cellular antioxidant defence^13^. These data suggest that A-T cells would show increased sensitivity to oxidative stress but this was not directly demonstrated until recently. In addressing this, we reported that airway epithelial cells isolated from a small number of patients with A-T showed increased sensitivity to oxidative stress induced by H_2_O_2_ or *S. pneumoniae* infection^23^. We hypothesised that recurrent infection with microorganisms producing H_2_O_2_ would induce oxidative damage *in vivo* and contribute to pulmonary complications in these patients. In the present study, we describe the microbial profile of the upper respiratory tracts of patients with A-T where we identified 20 major families including *Streptococcaceae*. Submerged cultures of immortalized respiratory cell lines and primary cells are commonly used as model systems to investigate respiratory disorders^24^. However, data generated using these models are not directly applicable to the human respiratory system since these cells fail to undergo mucociliary differentiation which is a key feature of the respiratory epithelium^25^. Hence there is a need for a more physiologically relevant model that recapitulates this. Consequently, we differentiated primary airway epithelial cells derived from patients with A-T and healthy controls to generate air-liquid interface (ALI) culture that mimic the conditions found in the human airways. In this study, we describe the effects of *S. pneumoniae* infection in differentiated ALI cells from patients with A-T of and investigated the mechanism of cell killing after infection of these cells.

## Results

### Microbial profiles of upper respiratory tracts from healthy controls and patients with A-T

**Figure 1:**
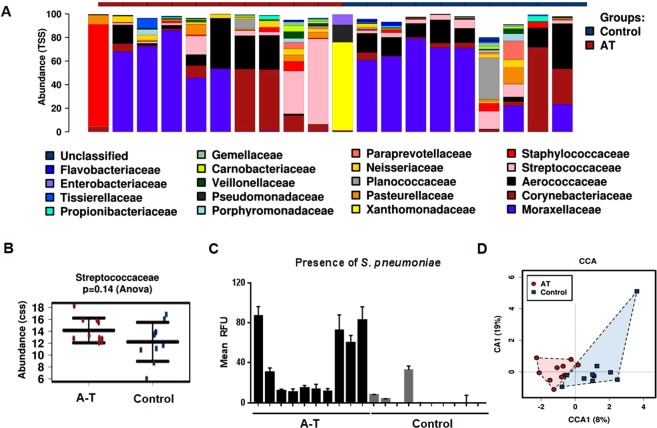
Difference in microbial profile of upper respiratory tracts between healthy controls and patients with A-T. 16S rDNA sequencing from nasal swabs obtained from patients with A-T (n = 10) and healthy controls (n = 10). (**A**) Distribution of the 20 most abundant bacterial families found in A-T and controls. (**B**) Rank test plot showed no significant differences (Anova p = 0.14) at this level in the *Streptococceae* family between A-T and controls. (**C**) PCR analysis revealed the presence of *S. pneumoniae* in all A-T samples. RFU; relative fluorescence units. (**D**) Multivariate analysis using canonical correlation analysis (CCA) showed distinct clustering of microbial populations between patients with A-T and healthy controls (p = 0.034).

The respiratory status and recent management of patients with A-T employed in this study is outlined in Table 1. The top twenty most abundant bacterial families detected in the upper airway are shown in Fig. 1A. These include *Streptococcaceae*, *Staphylococcaceae*, and *Pasteurellaceae* families which are commonly found in both upper and lower respiratory tracts. In addition, species from these families have been previously cultured from patients with A-T^5,9^. Although no significant differences were detected in the *Streptococceae* family between A-T and healthy control samples (Fig. 1B), the presence of *S. pneumoniae* was detected by PCR in all ten patients with A-T and was largely undetected in controls being evident at this level of detection in only three healthy controls out of ten (Fig. 1C). Multivariate analysis using canonical correlation analysis (CCA) also revealed a trend (p = 0.031, Adonis) for a distinctive microbial clustering pattern for patients with A-T as compared to healthy controls (Fig. 1D). In accordance with these observations, a support vector machine evaluated by leave-one-out cross-validation based on bacterial operational taxonomic units (OTU) was able to discriminate between the microbiota of A-T patients and healthy controls with 80% accuracy, indicating a substantially different microbiome composition in the upper respiratory tracts of patients with A-T as compared to healthy controls.

**Table 1:** Participant characteristics.

Participant	Sex	Age (y)	Current respiratory symptoms	Respiratory history	Respiratory treatment
1	M	2.7	Cough	Recurrent LRI	Low-dose amoxicillin clavulinate
2	M	6.8	Nil	Nil	Nil
3	F	5.1	Nil	Nil	Nil
4	F	12.7	Nil	Restrictive flow-volume loop	Nil
5	F	12.2	Nil	Past aspiration	Nil
6	F	8.5	Cough	Allergic rhinitis	Nil
7	M	13.5	Nil	Nil	Azithromycin; Dexamethasone
8	M	15.3	Nil	Previous recurrent pneumonia	Prednisolone
9	F	5.6	Nil	Minimal wet cough, 3 exacerbations in last 12 months.	Salbutamol; fluticasone propionate
10	F	4.8	Crackles	Gets prolonged chesty episodes - mostly dry barking cough and rhinorrhoea	Amoxicillin clavulinate

LRI: lower respiratory illness.

### Nasal airway epithelial cells from patients with A-T are more sensitive to *S. pneumoniae* infection

**Figure 2:**
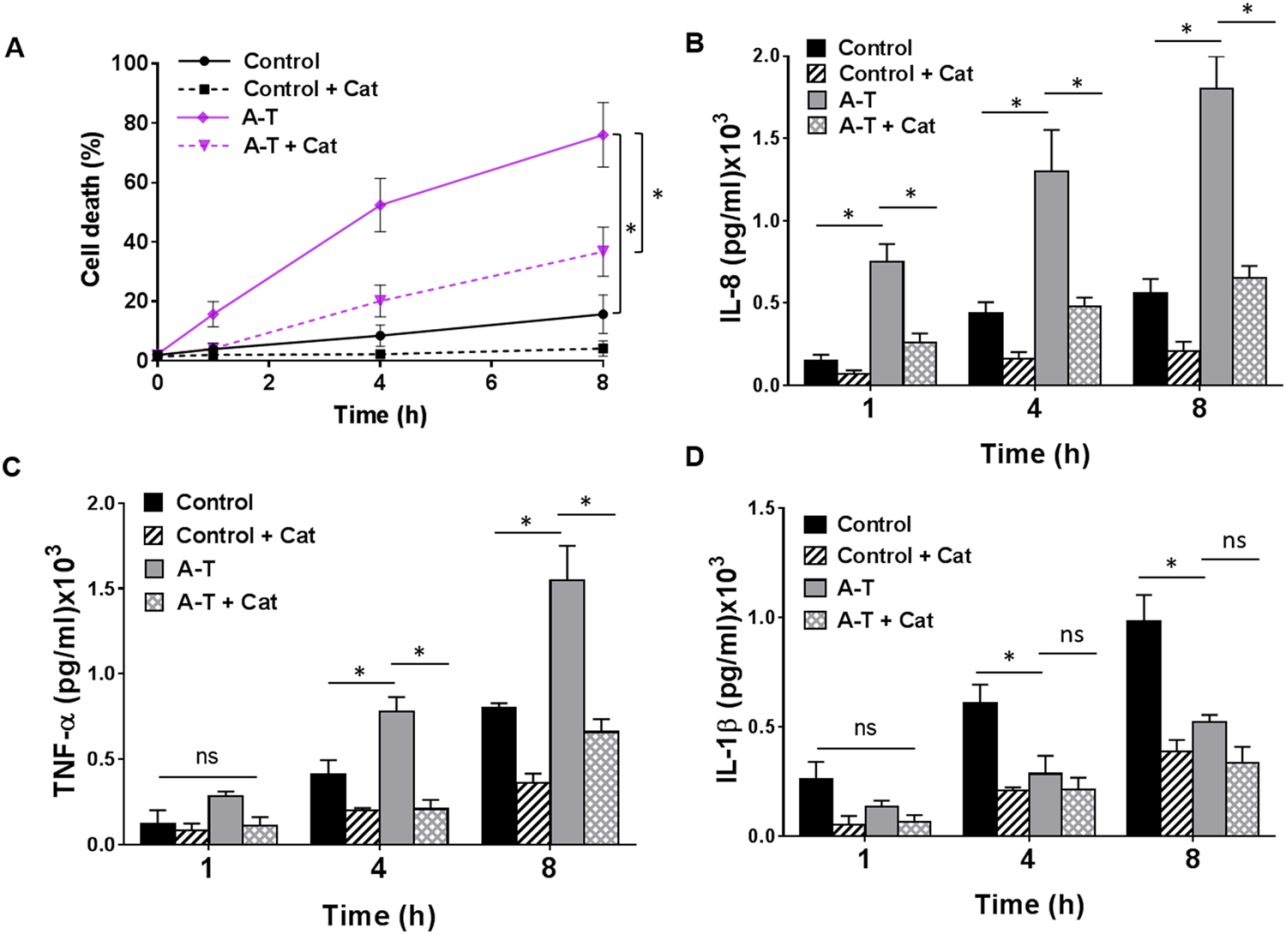
Increased sensitivity in A-T airway epithelial cells to *S. pneumoniae* infection. (**A**) Percentage of cell death induced by *S. pneumoniae* infection in control and A-T cells in the presence or absence of catalase (cat). Unpaired, two-tailed Mann-Whitney tests were carried out. *p < 0.03 between control and A-T at 8 h, and A-T and A-T + cat at 8 h. (**B**,**C**) Increase in inflammasome-independent pro-inflammatory cytokines IL-8 and TNF-α respectively in A-T as compared to control cells. Co-culture with catalase (cat) decreased this response. (**D**) Decrease in inflammasome-dependent cytokine IL-1β in A-T as compared to control cells, suggesting a defect in inflammasome activation. A-T n = 7, healthy controls n = 7. All data were plotted as the mean ± s.d. Kruskal-Wallis tests were performed and when significant (p < 0.05), Mann-Whitney tests were carried out. ns; not significant, *p < 0.03.

As reported in our initial study^23^, airway epithelial cells cultured from nasal scrapings from three patients with A-T exhibited increased sensitivity to oxidative stress as compared to that in healthy controls. Our present study extends this to submerged cultures derived from seven age-matched healthy controls and seven patients with A-T. These were infected with *S. pneumoniae* at MOI 100 and observed over a period of 8 h. To investigate whether oxidative stress induced by H_2_O_2_ production by *S. pneumoniae* is involved in cell killing, infected cells remained untreated or were exposed to catalase, an enzyme that catalyzes the degradation of H_2_O_2_ to water and oxygen^26^. Cell killing induced by *S. pneumoniae* was first assessed using the TUNEL assay. In the absence of catalase, ~15% cell death was observed in healthy controls as compared to ~76% for patients with A-T at 8 h (Fig. 2A), demonstrating an exquisite sensitivity of the A-T cells to infection. In the presence of catalase, the rate of cell death was significantly reduced in both healthy controls (~4%) and in patients with A-T (~36%) at 8 h, indicating that H_2_O_2_ produced by *S. pneumonia* plays a major role in the killing observed. To demonstrate that the increased killing in A-T cells was not due to an increase in bacterial numbers, we enumerated culture supernatants from control and A-T cells at 1, 4 and 8 h for *S. pneumoniae* numbers. There was no increase in number of bacteria in the cultures of cells obtained from A-T patients (Supplementary Table 1). We also used an additional control with heat-killed *S. pneumoniae* and observed a lesser effect on cell killing compared to the use of live bacteria (Supplementary Fig. 1A).

Persistent, low-grade inflammation has been suggested to be an underlying mechanism leading to the clinical pathogenesis of A-T^7,27^. We determined whether this inflammatory phenotype was observed in airway epithelial cells from patients with A-T. Levels of secreted IL-8 and TNF-α post-infection with *S. pneumonia* were significantly higher in submerged cultures derived from patients with A-T when compared to that from healthy controls (Fig. 2B,C). Similarly, the addition of catalase attenuated the elevation of these cytokines in both A-T and healthy controls, suggesting that alleviating the oxidative stress, resulting from *S. pneumoniae* infection, reduced the pro-inflammatory phenotype. IL-1β is a cytokine that is an important acute response factor of host defence against microbial infections and is also a key mediator of inflammation. It is present as a biologically inactive pro-IL-1β polypeptide in untreated cells that is post-translationally processed by Caspase-1 to generate the mature pro-inflammatory cytokine IL-1β^28^. It has also been reported that animals deficient in IL-1β are highly susceptible to microbial infections^29–31^. In contrast to the enhanced levels of the pro-inflammatory cytokines IL-8 and TNF-α, significantly lower levels of secreted IL-1β were observed in cultures derived from patients with A-T as compared to healthy controls post-infection with *S. pneumonia* (Fig. 2D). The IL-1β response was prevented by the specific NLRP3 inflammasome inhibitor MCC950 for both control and A-T cells (results not shown). Again in this case, exposure to heat-killed *S. pneumoniae* led to a lower level of induction of all three cytokines in both cell types (Supplementary Fig. 1B–D). These results for IL-1β suggest a defect in inflammasome activation in A-T cells following *S. pneumoniae* infection.

### Differentiated airway epithelial cell cultures derived from patients with A-T are more sensitive to infection with *S. pneumoniae*

**Figure 3:**
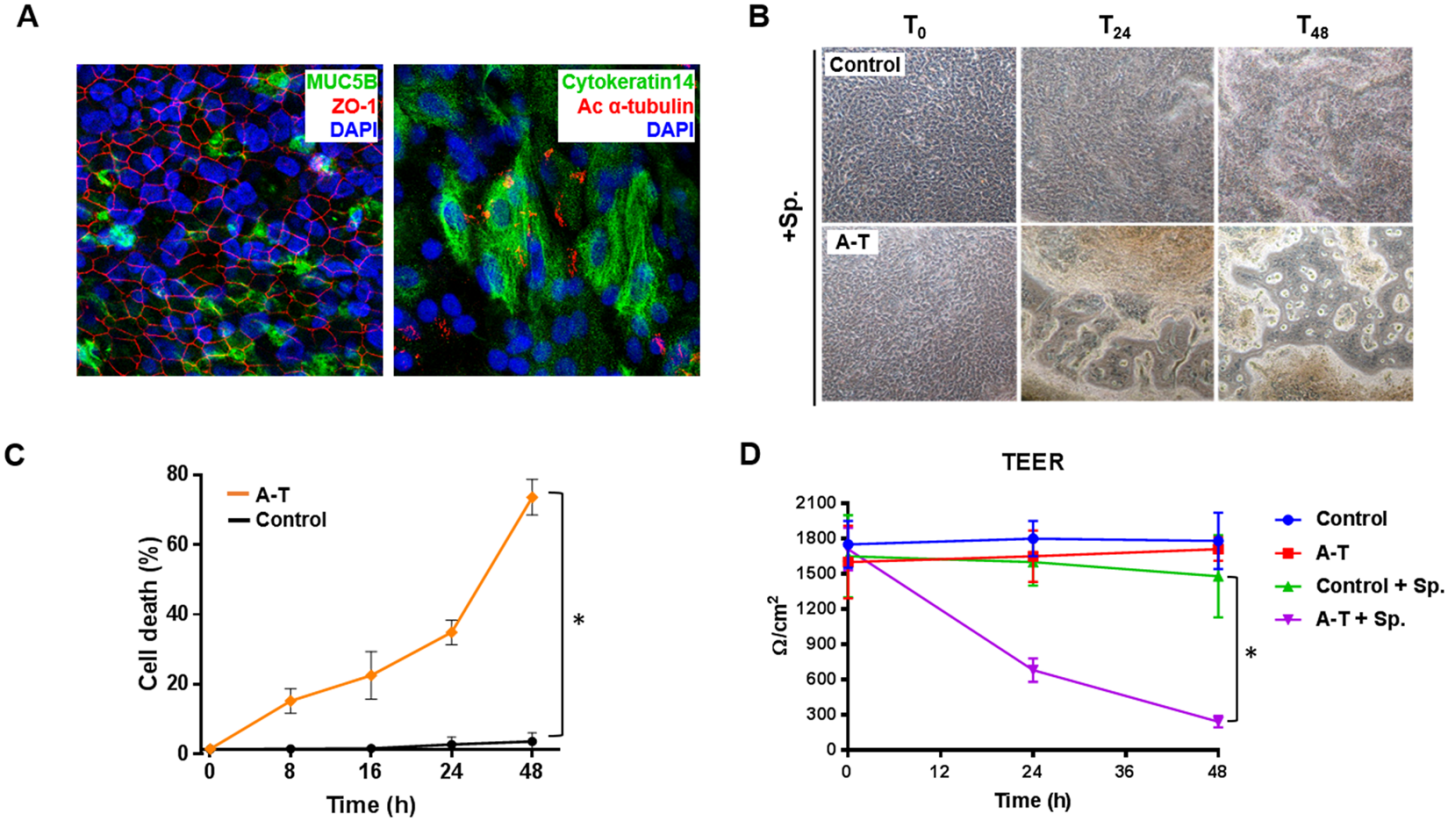
Increased sensitivity in A-T air-liquid interface cultures to *S. pneumoniae* infection. (**A**) Immunostaining of individual cell types in differentiated epithelia at ALI. Left panel: goblet cells (MUC5B; green), tight junctions (ZO-1; red); right panel: basal cells (cytokeratin 14; green) and ciliated epithelial cells (Ac α-tubulin; red). (**B**) Disruption of the respiratory epithelia at ALI by 24 h was observed in A-T but not in controls. (**C**) Percentage of cell death induced by *S. pneumonia* infection in control and A-T cells. (**D**) Transepithelial electrical resistance (TEER) reading performed on control and A-T ALI cultures post-infection. A-T n = 3, healthy controls n = 3. Unpaired, two-tailed Mann-Whitney tests were carried out. p < 0.03 between control and A-T at 48 h. All data were plotted as the mean ± s.d.

Many studies have used airway epithelial cells grown as submerged cultures to study cellular response to stressors, especially microbial infections^22,32,33^. While submerged cultures expand rapidly and are relatively easy to work with, they have the major disadvantage of not adequately representing the normal physiology of airway epithelial cells in the lung^34^. To overcome this limitation, primary airway epithelial cells are into well-differentiated cultures at an air-liquid interface (ALI) on a semi-permeable membrane^35^. This allows development of a well-differentiated pseudostratified polarized epithelium consisting of basal cells, goblet cell and ciliated epithelial cells^35,36^. We established differentiated ALI cultures from both healthy controls and patients with A-T which grew into well-differentiated airway epithelia resembling that of *in situ* lung tissue. Immunofluorescence confirmed the presence of the three main cell types of the native respiratory epithelium (ciliated, mucus-secreting goblet and basal cells) in the differentiated cultures using antibodies against acetylated α-tubulin, MUC5b and cytokeratin 14 respectively (Fig. 3A). The presence of a tight and intact epithelial layer was also confirmed by a positive staining for ZO-1 (tight junction protein-1) (Fig. 3A). Similar results were observed for cultures derived from healthy controls and patients with A-T. We have previously shown that submerged cultures of airway epithelial cells from patients with A-T are sensitive to *S. pneumoniae* infection compared to healthy controls^23^. To investigate whether this was recapitulated in differentiated ALI cultures, we performed this infection on these cultures and responses were observed over 48 h. Phase-contrast microscopy revealed that the cultures from healthy controls remained morphologically unchanged over the 48 h period. In contrast, the differentiated epithelial cell layer from patients with A-T showed signs of disruption by 24 h and this had increased in severity by 48 h (Fig. 3B). Cell killing induced by *S. pneumoniae* was first assessed using the TUNEL assay; <5% cell death was observed in healthy controls as compared to >70% of that in cells from patients with A-T (Fig. 3C). To further investigate the integrity of tight junction dynamics in the ALI cultures, transepithelial electrical resistance (TEER) measurements, a widely accepted quantitative technique used to assess barrier function in cells, were performed and results showed that those from healthy controls were not compromised (1650 ± 350 to 1480 ± 350 Ω/cm^2^) over the time course of infection. However, the readout from cultures derived from patients with A-T was significantly decreased by ~7-fold (1710 ± 180 to 240 ± 30 Ω/cm^2^) by 48 h (Fig. 3D).These results indicate that differentiated airway epithelial cells from patients with A-T are exquisitely sensitive to *S. pneumoniae* infection, reflecting increased lung susceptibility in patients with A-T.

### Dysregulation in inflammation response in differentiated airway epithelial cell cultures derived from patients with A-T following *S. pneumoniae* infection

**Figure 4:**
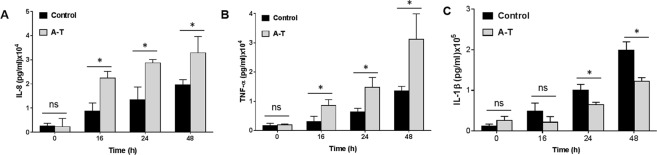
Increase in pro-inflammatory cytokines in A-T ALI cultures post-infection. (**A**,**B**) Increase in inflammasome-independent pro-inflammatory cytokines IL-8 and TNF-α in A-T as compared to control cells respectively. (**C**) Decrease in inflammasome-dependent cytokine IL-1β in A-T as compared to control cells, suggesting a defect in inflammasome activation. A-T n = 3, healthy controls n = 3. All data were plotted as the mean ± s.d. Unpaired, two-tailed Mann-Whitney tests were carried out. ns; not significant, *p < 0.03.

To determine whether dysregulation of the inflammation response was also occurring in air-liquid interface cultures derived from patients with A-T following *S. pneumoniae* infection, levels of secreted cytokines were assayed with AlphaLISA using wash fractions from the apical chambers of ALI cultures. As in the case of submerged cultures, there was a significant increase in IL-8 and TNF-α secretion providing further evidence for a pro-inflammatory phenotype (Fig. 4A,B). We also observed a decrease in IL-1β secretion at 48 h post-infection (Fig. 4C) consistent with a defect in inflammasome activation.

### Loss of ATM leads to a defect in inflammasome activation

**Figure 5:**
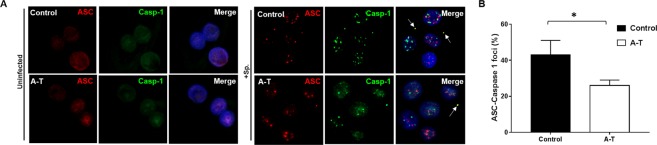
Loss of ATM impairs inflammasome activation following *S. pneumoniae* infection. (**A**) Immunostaining for caspase-1 (green) and ASC (red) showed evidence of inflammasome activation in control cells following infection with *S. pneumoniae*. In contrast, reduced levels of Caspase-1 and ASC specks were observed in A-T cells post-infection, suggesting a defect in inflammasome assembly. (**B**) Quantitation of Caspase-1-ASC foci formation in airway epithelial cells from healthy controls and patients with A-T. A-T n = 3, healthy controls n = 3. Experiment was performed in triplicates with a minimum of 200 cells per experiment counted for Caspase-1 and ASC foci. All data were plotted as the mean ± s.d. Unpaired, two-tailed Mann-Whitney test was performed, *p < 0.05.

Decreased secretion of IL-1β in airway epithelial cells from patients with A-T suggested a defect in inflammasome activation operating through the ASC/Caspase-1 signalling pathway. This has been reported previously in *Atm*-deficient mouse cells and in A-T human macrophages^37,38^. To further investigate the effect of *S. pneumoniae* infection on inflammasome activation the airway epithelial cells obtained from patients with A-T, we employed immunostaining using antibodies against ASC and Caspase-1 to study the formation of the inflammasome complex in these cells. As expected in uninfected cells, non-specific nuclear staining for ASC and Caspase-1 was observed in both cultures from healthy controls and patients with A-T. However, upon infection with *S. pneumoniae*, cultures derived from healthy controls showed ASC/Caspase-1 foci formation in ~45% of cells (Fig. 5A) whereas only ~25% of cultures from patients with A-T showed these foci (Fig. 5B), providing evidence that a defect at the level of inflammasome complex assembly occurs in the absence of ATM. This could explain the defect in IL-1β secretion where lower levels were observed in A-T cells infected with *S. pneumoniae*. It is also evident that ASC foci appear in the nuclei of both cell types but to the same extent. This has been reported previously after exposure of mouse macrophages in response to very high doses of ionizing radiation^39^.

## Discussion

Diseases of the respiratory system cause significant morbidity and are a frequent cause of death amongst patients with A-T^5,6,40^. Lung disease often progresses with age and neurological decline^6^. Immunodeficiency is also a common characteristic of the A-T phenotype and a significant proportion of patients with A-T are prone to recurrent respiratory tract infections^6,8,41^. Infections are usually caused by viruses during the first two years of life and by common bacterial pathogens in later childhood, including *H. influenzae*, *S. pneumoniae*, *P. aeruginosa* and *S. aureus*^6,9^. The epithelium of the human respiratory tract provides the entry point and plays a key role in the host defence system against invading pathogens, particles and environmental pollutants^42,43^. Bacterial colonization of the upper airways may act as a reservoir for certain bacteria and can precede spread to the lower airways^44,45^. Therefore it is important to better understand the pathogenesis of these infections so as to develop improved strategies for patient management. Timely identification and treatment of respiratory infections may help preserve lung function in patients and protect them from progressive lung disease. Here, we described a significant difference between the microbial profiles of the upper respiratory tracts of 10 patients with A-T and 10 healthy age-matched controls. Clearly, greater numbers of patients are required to establish the significance of these observations. In addition, it will be of interest to determine whether this difference in profile is specific to A-T or whether it also applies in the case of other respiratory disorders such as cystic fibrosis. In a study conducted by Schroeder and Zielen^9^, it was reported that bacterial colonization observed in patients with A-T included *S. aureus*, *H. influenzae*, *P. aeruginosa* and *S. pneumoniae*. Interestingly, we showed here in our study that *S. pneumoniae* was detected in all ten patients with A-T and in four healthy controls, suggesting a potential for increased colonization of *S. pneumoniae* in patients with A-T. As is also evident from Fig. 1A, there appears to be an enrichment of the family *Pasteurellaceae* and an increased level of a member of this family, *H. influenzae* in A-T (p = 0.08). However, we did not find any differences for *Pseudomonas* (p = 0.202) and a trend for lower prevalence of *Staphylococcus* in A-T (p = 0.14).

In our previous report, we showed that airway epithelial cells from patients with A-T were exquisitely sensitive to H_2_O_2_, an agent produced by *S. pneumoniae*, capable of inflicting oxidative damage^23^. Here, we have extended our study to additional patients with A-T and confirmed that airway epithelial cells from these patients exhibited a ~5 fold increase in cell death in response to infection as compared to healthy controls, further supporting a susceptibility to *S. pneumonia* infection as a consequence of its capacity to induce oxidative damage. Lung injuries are frequent and can influence A-T prognosis, however, the role of ATM in airway epithelial cells remains unclear. While many studies have used airway epithelial cells grown as submerged monolayer cultures to study their response to various stressors^24,46–48^, these cultures lose much of the differentiated functionality that characterizes the epithelia of the upper airways^49^. Thus, it is important to establish a model system that mimics closely the *in vivo* conditions of the airway epithelium. Here, we established for the first time differentiated ALI cultures from patients with A-T. These cultures were defined by a well-differentiated pseudostratified epithelium with basal cells and functional ciliated cells interspersed amongst mucus-secreting (goblet) cells, thereby producing a highly representative airway model for future studies into respiratory diseases in patients with A-T. These cultures also demonstrated a similar sensitivity to oxidative damage induced by *S. pneumoniae* infection. There is evidence that systemic inflammation and oxidative stress contribute to the pathophysiology of lung disease in A-T^27^. McGrath-Morrow *et al*. also showed that serum levels of pro-inflammatory cytokines, IL-8 and IL-6, were elevated in the serum from patients with A-T and suggested that markers of systemic inflammation may be useful in identifying individuals with A-T at increased risk for lower lung function^7,27^. Here, we demonstrated that airway epithelial cells from patients with A-T not only displayed increased sensitivity to oxidative stress but also elevated levels of pro-inflammatory cytokines, IL-8 and TNF-α following infection with *S. pneumoniae*, which would also support a role for inflammation in the process. In this context, it is of interest that *S. pneumoniae* bacterial burden in the nasopharynx is a major determinant of neutrophil recruitment during otitis media and that these inflammatory cells contribute to the secretion of IL-8 and TNF-α^50^. Neutrophil killing of *S. pneumonia* is mediated through myeloperoxidase, an enzyme that generates reactive oxygen species which, given the increased sensitivity of A-T cells to oxidative damage, could lead to exacerbated tissue damage including that of the lung in patients.

In contrast to the changes in pro-inflammatory cytokines in A-T airway epithelial cells, we showed that secretion of IL-1β was decreased in infected A-T epithelial cells compared to controls in infected cells. This is consistent with that observed from ATM-deficient monocyte-derived macrophages primed with LPS prior to *S. pneumoniae* infection^37^. In that study, the authors showed that ATM kinase activity was essential for inflammasome activation. Impaired processing of pro-IL-1β by Caspase-1 was observed and macrophages had significantly fewer ASC and Caspase-1 foci, pointing to a defect at the level of the inflammasome complex assembly. This defect was not confined to infection with *S. pneumoniae* but was also observed after infection with *P. aeruginosa, S. typhimurium* or after stimulation with ATP or nigericin exposure^37^. In the present study we showed that A-T airway epithelial cells were also defective in ASC-Caspase-1 complex assembly with cytoplasmic foci detected in only 25% of infected cells, which was approximately 50% of that observed in infected control airway epithelial cells. Reduced inflammasome activation in cells from A-T patients in response to S. pneumoniae could explain some of the hypersensitivity to infection. In addition to DNA damage from H2O2 exposure post-infection, it has also been shown that pneumolysin, a virulence factor produced by S. pneumoniae, causes both DNA damage^22^ and also inflammasome-dependent caspase-1 activation^51^. Mice lacking ASC show greater susceptibility to *S. pneumoniae* infection and impaired secretion of both IL-1β and IL-18^51^. We also observed the presence of ASC foci in nuclei in both control and A-T cells which was not previously observed in infected macrophages^37^. To our knowledge, these ASC foci have only been observed in HEK cells exposed to very high doses of radiation (80 Gy) and co-localized with AIM2, suggesting that nuclear-localized foci may also have some role in inflammasome activation^39^.

The model system we have established represents an excellent surrogate for investigating lung disease and can be employed not only for infection but also for screening drugs and environmental pollutants on an individualized basis. It has enabled us to pursue the hypothesis that oxidative stress arising from infection with specific microorganisms contributes to the development of lung disease in patients with A-T. Our results reveal that *S. pneumoniae* infection contributes to cell death in A-T cells by producing hydrogen peroxide to which these cells are particularly vulnerable. In addition, infection is associated with elevated secretion of pro-inflammatory cytokines which have the potential to cause tissue damage and is consistent with evidence for an inflammatory phenotype in patients with A-T^7,27,40^. Finally, we have shown that infection of airway epithelial cells exposes a defect in inflammasome activation which would also contribute to delays in clearing the burden of infection. The description of vulnerability to infection at different levels may facilitate the identification of new targets for intervention and the potential for more directed treatment for lung disease in patients with A-T. Identifying risk factors associated with respiratory complications will allow for timely treatment and improved outcomes. This study has the potential to present new opportunities for prevention of this debilitating aspect of this life threatening disorder.

## Methods

### Subjects and specimen collection

This study was approved by the Human Research Ethics Committees of the University of Queensland and Child Health Research Centre (HREC/09/QRCH/103). Parents of eligible participants gave informed consent for their child to be recruited for this study). Twenty children, ten with A-T (2.7 to 12.7 years, 5 females) and ten healthy age-matched controls (3 to 10.8 years, 2 females), were enrolled in this study. Each participant provided nasal samples for bacterial 16S ribosomal DNA sequencing and the nasal passage of each participant was swabbed gently using sterile nasal swabs (Copan, CA, USA). Scrapings from the inferior turbinate to establish nasal epithelial cultures were collected using a purpose-designed curette (ASI Rhino-Pro, Arlington Scientific, USA). Information on the respiratory status of patients with A-T is recorded in Table 1. All experiments were performed in accordance with relevant guidelines and regulations.

### DNA extraction and 16S ribosomal RNA sequencing

Genomic DNA from nasal swabs was extracted using the QIAamp DNA Mini Kit (Qiagen, Hilden, Germany) according to the manufacturer’s protocol. High-throughput sequencing of the V3-V4 hypervariable region of the bacterial 16S ribosomal RNA gene was performed on an Illumina MiSeq platform (Illumina, CA, USA) by the Australian Genome Research Facility (AGRF), Brisbane, on the DNA extracted from nasal swabs. Sequence data was analysed using QIIME. Generated OTU tables were then visualized and statistically analysed using the Calypso software^52^. Data was CSS normalized and differentially abundant taxa identified by Anova and ALDEx2, which accounts for the compositional characteristics of sequencing data. P-values were corrected for multiple testing using the Benjamini-Hochberg procedure. Principal components analysis (PCA) was run on OTU profiles and significance of clustering was estimated by Adonis using the Bray-Curtis dissimilarity.

### Submerged culture of primary airway epithelial cells

Primary airway epithelial cultures were established by seeding freshly collected nasal airway epithelial cells in bronchial epithelial growth medium (BEGM) containing the recommended supplements (BEGM BulletKit; Lonza, Basel, Switzerland) and 100 U/ml penicillin/streptomycin (Thermo Fisher Scientific, MA, USA) as described in Yeo *et al*.^23^. Cells were maintained in a humidified incubator at 37 °C with 5% CO_2_. The culture medium was then replaced with steroid- and antibiotic-free media 48 h prior to infection with *S. pneumoniae*.

### Air-liquid interface (ALI) cultures

5 × 10^4^ primary nasal airway epithelial cells were seeded into 6.5 mm transwell polyester membrane inserts with 0.4 µm pore size (Corning Inc, NY, USA) and grown to confluence in BEGM. Media from the apical chamber was subsequently removed and cells were maintained in differentiation medium (PneumaCult-ALI containing maintenance supplement, hydrocortisone, heparin as per manufacturer’s instructions; StemCell Technologies, Vancouver, Canada); and 100 U/ml penicillin/streptomycin (Thermo Fisher Scientific, MA, USA); fed through the basal chamber with the apical side of the epithelial cells exposed to air. Cells were cultured in ALI conditions for a minimum of 21 days or until differentiated (mucus production, presence of cilia beating as observed under light microscopy, increase in trans-epithelial resistance measurement (>1000 Ω/cm^2^). Differentiation was also confirmed using immunofluorescence for markers of these different cell types.

### Infection with Streptococcus pneumoniae

Nasal airway epithelial cells, grown to 80% confluence, were exposed to *S. pneumonia* (D39) at a multiplicity of infection (MOI) of 100. *S. pneumoniae* were cultured in Todd-Hewett broth (Oxoid, UK) to log phase (OD = 0.25). *S. pneumoniae* were washed using HBSS and an OD 1.1 suspension of *S. pneumoniae* was prepared in HBSS (previously determined to contain 4 × 10^8^ CFU/mL). The *S. pneumoniae* was diluted accordingly to infect submerged and ALI cultures at a MOI of 100. Each inoculum was serially diluted, plated onto horse blood agar plates and the colonies counted to confirm that an MOI of 100 was achieved. Uninfected control replicates were exposed only to cell culture medium. Submerged and ALI cultures were incubated with bacteria for 1 h and 3 h respectively at 37 °C, 5% CO_2_ and the media replaced with steroid- and antibiotic-free BEGM or PneumaCult-ALI. In submerged cultures of airway epithelial cells, bacterial infection was performed ± catalase (Sigma-Aldrich, MI, USA) at 1 mg/ml. To enumerate bacterial numbers following infection, the submerged infection experiment was carried out. 2.70E6 CFU were applied to cells at the beginning of the infection. Cells were infected for one hour at 37 °C, the inoculum was removed and fresh media was added to the cells (no antibiotics were used). Wells were designated for processing at either 1, 4 or 8 h. When the cells were processed at each respective time point, the bacteria was enumerated in the supernatants of the cultures.

### Immunofluorescence

Immunofluorescence was performed as described in Yeo *et al*.^53^. Briefly, cells were fixed using 4% paraformaldehyde and blocked in 20% fetal calf serum, 2% bovine serum albumin (BSA), 0.2% Triton X-100 in 1× PBS for 1 h at room temperature. Cells were incubated overnight at 4 °C in a humidified chamber with the following primary antibodies: mouse monoclonal anti-caspase-1 (sc-392736; Santa Cruz Biotechnology, TX, USA), rabbit polyclonal anti-ASC (ab180799; Abcam, Cambridge, UK), rabbit polyclonal anti-MUC5B (HPA008246; Sigma Aldrich, MI, USA), rabbit polyclonal anti-ZO1 (40–2200; Thermo Fisher Scientific; MA, USA), guinea pig polyclonal anti-cytokeratin 14 (ab192694; Abcam; Cambridge, UK) and mouse monoclonal anti-acetyl α-tubulin (32–2700; Thermo Fisher Scientific; MA, USA). Cells were subsequently washed thrice with 1 X PBS and incubated for 1 h at 37 °C in a humidified chamber with the following Alexa-Dye-conjugated secondary antibodies (Life Technologies, CA, USA) diluted 1:250: Alexa 488 chicken anti-mouse (A-21200), Alexa 594 donkey anti-rabbit (A-11032) and Alexa 488 goat anti-guinea pig (A-11073). Following, cells were washed thrice and Hoechst 33342 (1:10 000) (Life Technologies, CA, USA) was applied for 10 min to stain nuclei. Images were captured using a digital camera (AxioCam Mrm; Carl Zeiss Microimaging Inc., Jena, Germany) attached to a fluorescent microscope (Axioskop 2 Mot Plus; Carl Zeiss Microimaging) equipped with the appropriate filters, and the AxioVision 4.8 software (Carl Zeiss Microimaging Inc.).

### TUNEL Assay

TUNEL Assay was used to measure cell death and was performed using the fluorescence *in situ* cell death detection kit (Roche, Basel, Switzerland) as per the manufacturer’s protocol. Slides were visualized on the Axioskop 2 Mot Plus fluorescent microscope (Carl Zeiss Microimaging Inc., Jena, Germany), images were captured on the AxioCam Mrm digital camera (Carl Zeiss Microimaging Inc, Jena, Germany) and analysed using the AxioVision 4.8 software (Carl Zeiss Microimaging Inc, Jena, Germany).

### Transepithelial electrical resistance (TEER) measurement

1XPBS of 100 µl volume was added to the apical chamber of fully differentiated air-liquid interface cultures. Trans-epithelial electrical resistance (TEER) was then determined using the EVOM2 epithelial voltohmmeter (World Precision Instruments, FL, USA) equipped with the STX2 electrodes and a mean resistance was calculated. Values were then corrected for fluid resistance (insert with no cells) and surface area. All measurements were performed in triplicate.

### Cytokine quantitation

Secreted cytokines were measured from cell culture supernatant. For submerged cultures, supernatants were collected 0, 1, 4 and 8 h post-infection. For ALI cultures, 100 µl of PBS was applied to the apical chambers of inserts and wash fractions were collected at 0, 16, 24 and 48 h post-infection. IL-8, TNF-α and IL-1β were quantitated using AlphaLISA according to the manufacturers’ protocol (Perkin Elmer, MA, USA).

### Statistical analysis

Data are presented as mean ± SD. To determine differences between treatment groups, Kruskal-Wallis tests were performed. Following a result of p < 0.05, unpaired Mann-Whitney tests were carried out. Statistical analyses were performed using the GraphPad Prism 7 software (GraphPad Software Inc., CA, USA).

## Supplementary information

Supplementary Material

## Data Availability

The datasets generated during and/or analysed during the current study are available from the corresponding author on reasonable request.
