# Author Correction: Increased susceptibility of airway epithelial cells from ataxia-telangiectasia to *S. pneumoniae* infection due to oxidative damage and impaired innate immunity

**DOI:** 10.1038/s41598-020-69649-w

**Published:** 2020-07-24

**Authors:** Abrey J. Yeo, Anna Henningham, Emmanuelle Fantino, Sally Galbraith, Lutz Krause, Claire E. Wainwright, Peter D. Sly, Martin F. Lavin

**Affiliations:** 1 Neuroscience & Infectious Disease Group, The University of Queensland Centre for Clinical Research, Herston, Queensland Australia; 2 Children’s Lung, Environment and Asthma Research (CLEAR) Group, Child Health Research Centre, The University of Queensland, South Brisbane, Queensland Australia; 3 The University of Queensland Diamantina Institute, Translational Research Institute, Woolloongabba, Queensland Australia; 4 Lady Cilento Children’s Hospital, South Brisbane, Queensland Australia

Correction to: *Scientific Reports* 10.1038/s41598-019-38901-3, published online 22 February 2019

The Acknowledgements section in this Article is incomplete.

“We would like to acknowledge Avril Robertson, The University of Queensland, for providing the supply of MCC950. We also acknowledge that this work was supported by BrAshA-T, Australia (RM2016001474), the A-T Children’s Project, USA (RM2016000563) and the National Health and Medical Research Council, Australia (GNT1020028).”

should read:

“We would like to acknowledge Avril Robertson, The University of Queensland, for providing the supply of MCC950. We also acknowledge that this work was supported by BrAshA-T, Australia (RM2016001474), the A-T Children’s Project, USA (RM2016000563), National Health and Medical Research Council, Australia (GNT1020028) and the Children’s Hospital Foundation (RM2018002270).”

