# Supplementary material for: Increased susceptibility of airway epithelial cells from ataxia-telangiectasia to S. pneumoniae infection due to oxidative damage and impaired innate immunity
Source: Sci Rep. 2019 Feb 22;9:2627. doi: 10.1038/s41598-019-38901-3 (PMC6385340; doi:10.1038/s41598-019-38901-3)
Supplement: Supplementary file 1 — Supplementary Material [file 41598_2019_38901_MOESM1_ESM.docx]

**Increased susceptibility of airway epithelial cells from ataxia-telangiectasia to *S. pneumoniae* infection due to oxidative damage and impaired innate immunity**

Abrey J. Yeo^1^, Anna Henningham^2^, Emmanuelle Fantino^2^, Sally Galbraith^2^, Lutz Krause^3^, Claire E. Wainwright^2,4^, Peter D. Sly^2^, Martin F. Lavin^1,*^

^1^ Neuroscience& Infectious Disease Group, The University of Queensland Centre for Clinical Research, Herston, Queensland, Australia

^2^ Children's Lung, Environment and Asthma Research (CLEAR) Group, Child Health Research Centre, The University of Queensland, South Brisbane, Queensland, Australia

^3^The University of Queensland Diamantina Institute, Translational Research Institute, Woolloongabba, Queensland, Australia

^4^Lady Cilento Children’s Hospital, South Brisbane, Queensland, Australia

*****Corresponding Author: Martin F. Lavin. Cancer and Neuroscience, the University of Queensland Centre for Clinical Research, Building 71/918 RBWH, Herston, Brisbane, Australia. E-mail: m.lavin@uq.edu.au

**Supplementary Material**


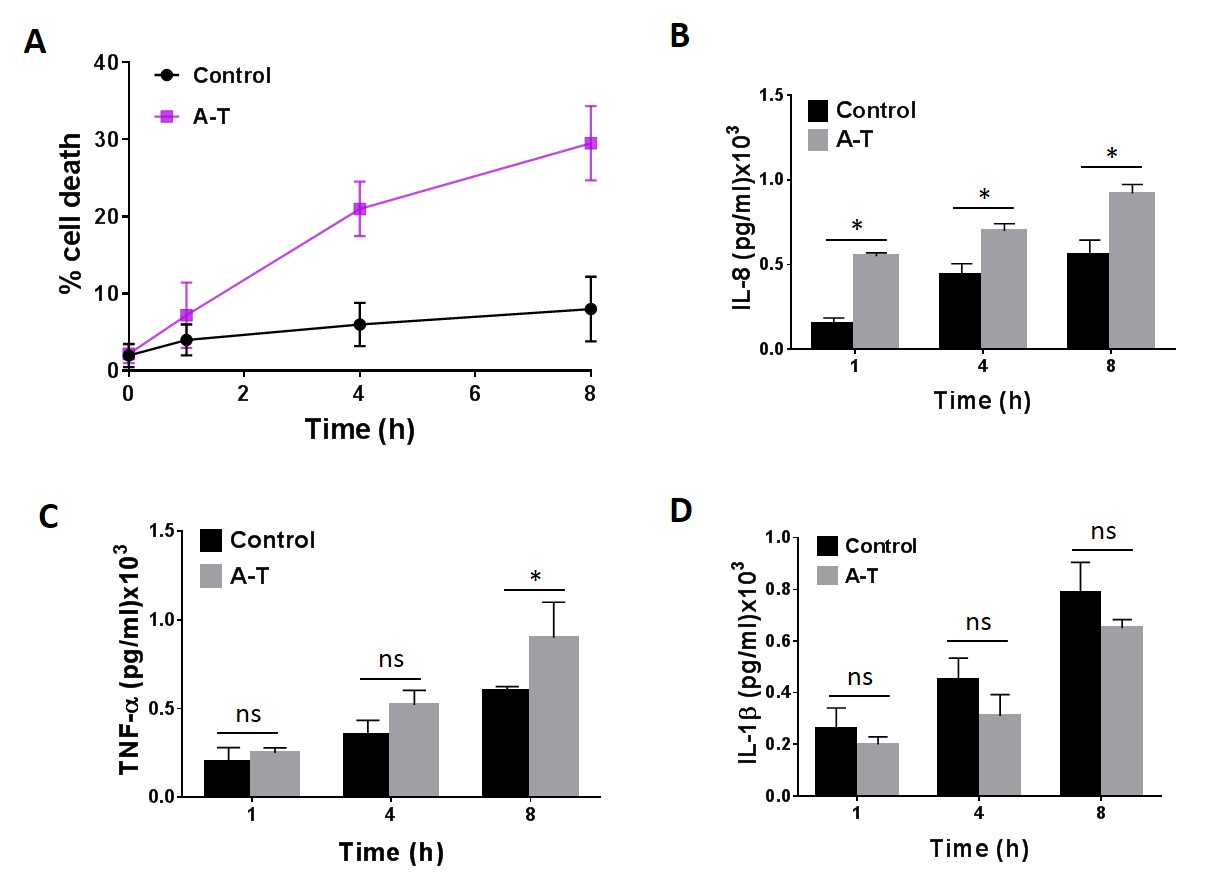


**Supplementary Figure 1:** **Sensitivity in A-T airway epithelial cells to heat-killed *S. pneumoniae* infection.**

**A.** Percentage of cell death induced by heat-killed *S. pneumoniae* infection in control and A-T cells. **B and C.** Moderate increase in inflammasome-independent pro-inflammatory cytokines IL-8 and TNF-α respectively in A-T as compared to control cells following exposure to heat-killed *S. pneumoniae*. **D.** Moderate induction of inflammasome-dependent cytokine IL-1β in control and A-T cells. A-T n=2, healthy controls n=2. All data were plotted as the mean ± s.d of three separate experiments. Unpaired, two-tailed Mann-Whitney test was performed. ns; not significant, *p<0.05.

| **Time Point** | **Control** | **A-T Patient** |
| --- | --- | --- |
| 1 h | 1.2 x 10E6 | 1.5 x 10E6 |
| 4 h | 1.5 x 10E6 | 1.5 x 10E6 |
| 8 h | 2.1 x 10E6 | 2.4 x 10E6 |

**Supplementary Table 1:** Enumeration of bacterial numbers from supernatant from control and A-T cells at 1, 4 and 8h post-infection.
